# Neurocognitive, Social-Cognitive and Quality of Life Differences in Children with Advanced Heart Failure: A Single-Center Cross-Sectional Study

**DOI:** 10.3390/children13081083

**Published:** 2026-08-15

**Authors:** Didem Celik, Aysen Yaprak Kapkin, Nazli Burcu Ozbaran, Nagehan Demiral, Zulal Ulger Tutar, Umit Kahraman, Osman Nuri Tuncer, Ahmet Daylan, Yüksel Atay, Çağatay Engin, Tahir Yagdi, Mustafa Ozbaran

**Affiliations:** 1 Child and Adolescent Psychiatry, Ege University Faculty of Medicine, 35100 Izmir, Turkey; 2 Cardiovascular Surgery, Ege University Faculty of Medicine, 35100 Izmir, Turkey; 3 Pediatric Cardiology, Ege University Faculty of Medicine, 35100 Izmir, Turkey

**Keywords:** pediatric heart failure, heart transplantation, ventricular assist device, neurocognitive function, social cognition, quality of life, psychiatric symptoms, executive functions

## Abstract

**Highlights:**

**What are the main findings?**

• Decreased global cognition (WISC-IV), social cognition (Eyes Test), health-related quality of life (both child and parent), higher parent-reported anxiety, and similar child-reported anxiety.
• No cognitive differences among the LVAD/transplant/waiting groups.

**What are the implications of the main findings?**

• Parent and child self-reports provide complementary information.
• Neurocognitive vulnerability exists in all heart failure patient groups. Healthcare should be planned considering the neurocognitive and social development.

**Abstract:**

Background: Children with heart failure (HF) are at risk of cognitive and psychiatric problems, and reduced health-related quality of life (QoL), yet data remain limited. This study compared neurocognition, social cognition, health-related quality of life, and psychiatric symptoms among children awaiting transplantation with or without ventricular assist devices (VADs), those who had undergone heart transplantation, and matched controls. Methods: Twenty-three patients at Ege University and 17 age- and sex-matched controls were assessed using the Schedule for Affective Disorders and Schizophrenia for School-Aged Children, Present and Lifetime Version (K-SADS-PL), Screen for Child Anxiety-Related Emotional Disorders (SCARED), Children’s Depression Inventory (CDI), Wechsler Intelligence Scale for Children–Fourth Edition (WISC-IV), Eyes Test, and Pediatric Quality of Life Inventory (PedsQL), evaluating psychiatric diagnoses, anxiety and depressive symptoms, cognition, social cognition, and health-related quality of life. Results: Diagnosis rates and CDI and SCARED child scores were comparable, but SCARED parent scores were significantly higher in the HF group. Patients scored significantly lower on most WISC-IV subtests (8 of 10) and all composite scores and the Eyes Test, indicating impaired social cognition. Child- and parent-reported PedsQL scores were significantly lower across all domains. Cognitive outcomes did not differ significantly across treatment subgroups, though these comparisons were underpowered. Conclusions: Children with HF show marked impairments in neurocognition, social cognition, and health-related quality of life versus healthy peers. Cognitive outcomes did not differ significantly across treatment subgroups, although these subgroup analyses (5–7 children per group) were underpowered and cannot establish the absence of a treatment effect. These findings underscore the need for integrated multidisciplinary care.

## 1. Introduction

Children with congenital heart disease are at increased risk of delays in cognitive development and psychological functioning [1,2], but data specific to heart failure (HF) remain limited. It has been shown that the children receiving only medical therapy are at risk for anxiety and depression like children with congenital heart disease [3]. In a previous report, we found similar results in children supported with ventricular assist devices (VADs) [4].

Reduced cerebral perfusion, cardiac arrests, surgery, hospitalizations, and separation from school and social life; such mechanisms have been described largely in adults with HF [5,6].

Despite growing interest, data remain scarce. The relationship between cognitive functioning, social cognition, and health-related quality of life has not been investigated in this population.

Social development is as crucial as neurocognitive development for daily life. Therefore, besides neurocognitive tests, social cognition tests were administered in this trial. To our knowledge, this is the first study evaluating social cognition by the Eyes Test in children with HF. Children with end-stage HF were compared with healthy controls to investigate the impact of HF on cognitive functioning, psychological status, social cognition, and health-related quality of life.

Aims and hypotheses: The present study aimed to characterize neurocognitive functioning, social cognition, psychiatric morbidity, and health-related quality of life in children and adolescents with advanced (end-stage) heart failure, and to compare them with age- and sex-matched healthy controls. Because the design was cross-sectional, comparisons across treatment stages were regarded as exploratory. We tested the following a priori hypotheses: (1) heart failure is associated with poorer neurocognitive functioning than in healthy controls; (2) psychiatric disorders, including depression and anxiety disorders, are more frequent in children with heart failure than in controls; (3) heart failure is associated with lower health-related quality of life; (4) among children with end-stage heart failure, those who have undergone heart transplantation show better cognitive functioning than those awaiting transplantation with ventricular assist device support (examined as an exploratory, underpowered subgroup comparison); (5) heart failure is associated with poorer social cognition; and (6) within a given treatment subgroup, children with better cognitive functioning also report higher quality of life.

## 2. Materials and Methods

This is a single-center, cross-sectional, prospective, exploratory study that was executed with the co-operation of the Departments of Child and Adolescent Psychiatry, Pediatric Cardiology, and Cardiovascular Surgery. The study was conducted in accordance with the Declaration of Helsinki and approved by the Institutional Review Board of the Ethical Committee of Ege University (decision number 20-2.1T/51—19 February 2020).

The patients, between the ages of 6 and 17 and on follow-up in the Department of Pediatric Cardiology and Cardiovascular Surgery, were included between March 2020 and January 2021. No participant had completed their 17th year at testing; all were aged 6 years 0 months to 16 years 11 months, which is within the normative range of the Turkish WISC-IV (6:0–16:11). The control group consisted of age- and sex-matched healthy volunteers. Controls were group-matched to patients on age and sex, not individually pair-matched, which accounts for the unequal group sizes. They were healthy volunteers with no known chronic medical, neurological, developmental, or learning disorder and were recruited over the same period (March 2020–January 2021) and assessed under the same conditions by the same team and underwent the identical psychiatric evaluation, so screening for psychiatric morbidity was equivalent across groups. The study took place in the COVID-19 pandemic era; hence, finding healthy controls was more challenging. The control group was recruited through an announcement circulated among faculty members. Children of faculty members who met the study’s eligibility criteria and whose parents provided informed consent were enrolled in the study.

Informed consent was obtained from all subjects involved in the study. All cases and their parents were included after the oral and written informed consents.

Children younger than 6 or older than 16 years 11 months of age, who were non-literate or in the postoperative first 3 months or in critical condition, and those without consent, were excluded.

A sociodemographic data form was applied for all cases.

A medical associate conducted the initial psychiatric evaluation. Following a subsequent visit by two academic members, a psychiatric diagnosis was made using the Turkish adaptation of the Diagnostic and Statistical Manual of Mental Disorders, Fifth Edition (DSM-5) [7,8].

Concomitant psychiatric disorders were evaluated by the Turkish adaptation of the Schedule for Affective Disorders and Schizophrenia for School-Aged Children, Present and Lifetime Version (K-SADS-PL) [8,9].

The K-SADS-PL was administered by the child and adolescent psychiatry resident who performed the initial evaluation, and each participant was then re-interviewed by two senior academic child and adolescent psychiatrists; final diagnoses were reached by consensus between them. Because diagnoses were established by consensus rather than by independent parallel ratings, formal inter-rater reliability was not separately calculated.

Afterwards, cognitive function was assessed with the Wechsler Intelligence Scale for Children–Fourth Edition (WISC-IV; Turkish standardization, normative range 6 y 0 mo–16 y 11 mo), administered and scored by trained clinical psychologists [10,11]. Nineteen cases from the patient group and all cases from the control group took the test. Complete composite (index) scores could be derived for 18 of these patients; one patient had insufficient completed subtests for composite computation, so the index-score analyses are based on 18 patients (individual subtest *n* ranged from 17 to 19). The WISC-IV was administered and scored by one of two trained clinical psychologists, and the Eyes Tests, together with the self- and parent-report questionnaires were completed within the same assessment session. Owing to the clinical setting, assessors were aware of the participants’ heart-failure status and treatment subgroup (the study was not blinded), which is acknowledged as a limitation.

For all the cases, the age-based Pediatric Quality of Life Inventory (PedsQL) Child Form [12,13,14] for evaluation of health-related quality of life, the Children’s Depression Inventory (CDI) [15,16] for evaluation of depressive symptoms, and the Screen for Child Anxiety-Related Emotional Disorders (SCARED) Child Form [17,18] for evaluation of anxiety symptoms were applied. For all families, Parent Forms of PedsQL and SCARED were applied. Additionally, social cognition was assessed with the child version of the “Reading the Mind in the Eyes” Test [19], comprising 28 photographs of the eye region; for each item, participants selected which of four words best described the person’s mental state (1 point per correct response; range 0–28), with higher scores indicating better mental-state recognition. The Turkish child form, validated by Girli, was used [20].

For the trial, 26 patients with HF were contacted: two of these patients were unable to undergo psychiatric evaluation due to significant aftereffects of cerebrovascular incidents that happened before visit dates, and one patient was deceased before the planned visit. A total of 23 end-stage HF patients were included.

For the control group that was planned to be paired according to age and sex, 17 volunteers between the ages of 6 and 17 were included.

## 3. Statistical Analysis

Numerical variables are summarized as mean ± standard deviation or median (minimum–maximum) and categorical variables as frequencies and percentages, using IBM SPSS Statistics for Windows, version 25.0 (IBM Corp., Armonk, NY, USA). Effect sizes, confidence intervals, and false-discovery-rate adjustments were computed in R version 4.3. Because pediatric end-stage heart failure is rare, the sample was small; no a priori power calculation was performed, and the study is presented as exploratory and hypothesis-generating. A two-sided *p* < 0.05 was considered statistically significant for the primary outcomes.

Normality was assessed separately in each comparison group with the Shapiro–Wilk test, acknowledging its limited power at these sample sizes. Two independent groups were compared with the independent-samples *t* test or the Mann–Whitney U test as appropriate; the three treatment subgroups were compared with the Kruskal–Wallis test, with Dunn’s test and Bonferroni adjustment for post hoc pairwise comparisons. Because the number of tests was large relative to the sample, a small set of primary outcomes was designated in advance—WISC-IV Full-Scale IQ, PedsQL Total Scale Score (child and parent proxy report), and the Eyes Test—and all other comparisons (individual anxiety and quality of life subdomains, WISC-IV subtests and subindices, treatment-subgroup contrasts, and all correlations) were treated as exploratory, with the Benjamini–Hochberg false-discovery-rate procedure applied within each exploratory family.

Categorical variables were compared with Fisher’s exact test, used in preference to the Pearson chi-square throughout because expected cell counts were frequently below five. Associations between neurocognitive measures and health-related quality of life scores were examined with Spearman’s rank correlation coefficient; given the small number of observations (*n* = 17–18), these correlations are unstable and are interpreted with caution. Standardized effect sizes with 95% confidence intervals are reported for the principal comparisons (Hedges’ g for continuous outcomes, rank-biserial correlation for Mann–Whitney comparisons, and ε^2^ for Kruskal–Wallis tests) rather than *p* values alone.

Primary comparisons are reported as unadjusted associations. Because the sample was too small to support a multivariable model containing several covariates, full adjustment was not attempted; instead, as a sensitivity analysis, the between-group difference in Full-Scale IQ was re-examined after adjustment for parental education using nonparametric rank analysis of covariance (Quade’s method) and within parental-education strata.

## 4. Results

A total of 40 participants were included in the study, comprising 23 children with end-stage HF (57%) and 17 age- and sex-matched healthy controls (43%). There were no significant differences between groups in age, sex, or school grade (Table 1). Parental educational levels were significantly lower in the HF group, and home-based education was more common among them.

Within patients, 34.8% (*n* = 8) were on VAD support, and 21.7% (*n* = 5) had undergone transplantation. The remaining were awaiting transplantation, most commonly due to dilated cardiomyopathy (*n* = 13, 56.5%) and restrictive cardiomyopathy (*n* = 6, 26.1%), followed by arrhythmogenic right ventricular dysplasia (*n* = 2, 8.7%), endocardial cushion defect (*n* = 1, 4.3%), and cardiomyopathy secondary to ectopic atrial tachycardia (*n* = 1, 4.3%). Detailed information on subgroup carachteristics are given in Table 2.

**Table 2:** Clinical characteristics of the three treatment subgroups.

Characteristic	Device-Supported, Awaiting Transplant (*n* = 8)	No Device, Awaiting Transplant (*n* = 10)	Post-Transplant (*n* = 5)
**Age, years—median (range)**	14 (8–16)	11 (7–17)	15 (14–17)
**Sex, male/female**	6/2	5/5	1/4
**Underlying cardiac diagnosis,** ***n***			
Dilated cardiomyopathy	7	2	3
Restrictive cardiomyopathy	0	4	2
Arrhythmogenic RV dysplasia	1	1	0
Endocardial cushion defect	0	1	0
CMP 2° ectopic atrial tachycardia	0	1	0
**Coexisting organic disorder,** ***n***	0	2 ^a^	2 ^b^

^a^ diaphragmatic hernia; inguinal hernia. ^b^ epilepsy; brain tumor with hypothyroidism. RV, right ventricular; CMP, cardiomyopathy; NYHA, New York Heart Association. Note: three additional patients were assessed but not enrolled—two with severe sequelae after a cerebrovascular event and one who died before the visit.

No statistically significant differences in psychiatric diagnoses were observed between groups.

Child-reported depressive and anxiety symptoms were comparable between groups. Parent-reported anxiety scores were significantly higher in the HF group (Table 3).

Quality of life was markedly reduced in children with HF. Both child- and parent-reported PedsQL scores were significantly lower across all domains (Table 4).

Social cognition, assessed by the Eyes Test, was significantly impaired in the HF group compared with controls (median 19 [8–24] vs. 21 [9–25]; *p* = 0.017; rank-biserial r = 0.44).

Neurocognitive assessment revealed substantially lower WISC-IV index scores in the HF group (Table 5a). When patients were stratified by treatment modality, no significant differences were observed across any cognitive indices (subgroup analyses are shown in Table 5a). Subtest scores are detailed in Table 5b.

Sensitivity analysis: the lower Full-Scale IQ in the heart-failure group persisted after adjustment for maternal education (Quade rank ANCOVA, *p* = 0.022) and paternal education (*p* = 0.002), and remained significant when the analysis was restricted to families in which the mother had no university education (heart-failure 71.7 vs. control 91.2; *p* = 0.015). The cognitive difference is therefore not explained by parental education alone, although residual confounding cannot be excluded in a sample of this size.

No significant associations were identified between WISC-IV scores and child-reported health-related quality of life. In contrast, parent-reported health-related quality of life demonstrated moderate correlations in the HF group (Table 6). Similar associations between working memory and multiple Health-related quality of life domains were also observed in the control group (Table 7). However, after Benjamini–Hochberg correction, none of the WISC–PedsQL correlations in Table 6 and Table 7 remained statistically significant.

## 5. Discussion

Impaired cognitive functioning in pediatric patients with congenital heart disease was demonstrated previously, whereas evidence in pediatric HF remains limited [21]. To the best of our knowledge, this is the first study evaluating social cognition using the Eyes Test in children with advanced HF.

Although we anticipated a higher psychiatric symptom burden in the HF group, rates of psychiatric diagnoses and child-reported depression and anxiety scores were comparable between groups. However, parents in the HF cohort rated their children as having significantly more anxiety symptoms on the SCARED-Parent form, which measures the parent’s report of the child’s symptoms rather than the parent’s own anxiety. This discrepancy between child and caregiver may reflect limited emotional awareness in children or increased parental vigilance while facing a life-threatening disease. Previous studies have repeatedly demonstrated this discrepancy [3,22,23]. Many studies have reported that parents underestimate their children’s psychosocial symptoms [22,24]; however, our finding contradicts this. We believe these findings may be related to parenting style and emotional reporting, which have been well-demonstrated and shown to be related to cultural characteristics [25,26,27]. Reviews of parent- and child-reported quality of life, based mainly on Western samples, indicate modest and domain-dependent agreement between informants [28]; by contrast, parents in some Asian communities underestimate their children’s distress relative to the children themselves [26]. Combined, these findings underline the importance of incorporating both caregiver and patient perspectives into the routine psychosocial assessment of pediatric patients with advanced HF.

This study provides the first objective evaluation of social cognition in children with advanced HF. Previous studies in children with congenital heart disease have shown that chronic illness is associated with impaired social cognition [29,30]. Consistently lower Eyes Test scores in the HF group point to impairments in the ability to recognize emotional cues, which may affect peer relationships and adaptive functioning [31]. This result underlines the need for detailed evaluation, including neurocognitive function and social relations, in routine follow-up.

Neurocognitive impairment is increasingly recognized as a common, clinically relevant comorbidity in HF. Studies in adults with heart failure suggest that 25% to 75% of patients with HF demonstrate measurable deficits in attention, executive function, processing speed, and memory, likely related to chronic cerebral hypoperfusion, systemic inflammation, and structural brain injury [6,32]. Cognitive vulnerability appears to be most pronounced in patients with unsupported end-stage HF, in whom reduced cardiac output and recurrent decompensations may exacerbate cerebral dysfunction in these adult cohorts. [6,32]. Mechanical circulatory support with left ventricular assist devices (LVAD) may partially reverse these deficits. In adult LVAD recipients, several longitudinal studies have demonstrated stabilization or improvement in global cognitive scores following LVAD implantation; whether this benefit extends to children is unknown. [32,33,34]. In adult heart transplant recipients, neurocognitive and quality of life outcomes are variable: although some recover as systemic hemodynamics improve, cognitive dysfunction persists in a subset, potentially related to perioperative neurological injury, immunosuppressive therapy, and pre-existing neurocognitive vulnerability [35]. Collectively, these findings—derived largely from adult populations—suggest a continuum. The greatest impairment was observed in unsupported HF, partial improvement after LVAD support, and variable but often incomplete recovery following heart transplantation [32,34,35]. This framework has not been established in children and is applied here only as a reference model. In children specifically, neurodevelopmental delay is common after heart transplantation in early childhood [36], and neurodevelopmental and psychosocial vulnerability is increasingly recognized in children supported with ventricular assist devices [37,38], although dedicated cognitive data in pediatric heart failure—rather than congenital heart disease—remain scarce, which our findings begin to address.

Interpreted against this adult-derived framework, children with HF in our cohort exhibited broadly reduced performance on the WISC-IV. All composite scores were also significantly lower in the HF group, indicating a global pattern of cognitive vulnerability. Cognitive outcomes were similar among HF subgroups, although, with only 5–7 children per subgroup, these analyses are underpowered and, in this cross-sectional design, cannot demonstrate cognitive decline or its persistence over time. Although concerns have been raised regarding cognitive decline in adults during LVAD support [33], our findings did not demonstrate worse cognitive performance in children on LVAD support compared with other HF subgroups. This pattern supports that pediatric HF is associated with neurocognitive vulnerability that may not be fully explained by treatment modality alone. Because the cohort was assessed at a single time point, these data cannot separate disease burden from treatment stage; a longitudinal study would be required to do so. Both child- and parent-reported health-related quality of life scores were significantly lower in the HF group, indicating generalized effects in physical, emotional, and social functioning, consistent with the reduced quality of life reported for pediatric heart transplant recipients [39]. This concordance between child self and caregiver proxy reports highlights the impact of end-stage HF on daily life and the need for integrated psychosocial support in addition to medical management.

In the present study, while child-reported health-related quality of life was not associated with cognitive performance, parent-reported measures showed significant correlations with social and school functioning, psychosocial health, and total scale scores. In the parent proxy reports of the HF group, verbal comprehension and working memory emerged as key correlates of school functioning, psychosocial health, and overall health-related quality of life. Controls also exhibited similar correlations between working memory and various health-related quality of life scores. These patterns highlight the importance of executive and verbal abilities for functioning; however, after correction for multiple testing, none of these correlations remained statistically significant, so they should be regarded as exploratory and do not establish that caregiver reports are more reliable than child self-reports.

Discrepancies between child self-reports and parent proxy reports are well documented in pediatric chronic illness and may reflect limited emotional insight, coping mechanisms such as denial, and the greater sensitivity of caregivers to functional impairment [28]. Along with these observations, parent-reported health-related quality of life measures in our cohort offered valuable complementary perspectives and should be interpreted alongside child self-reports. Accordingly, our hypothesis that children with better cognitive functioning within the same clinical subgroup would also report higher health-related quality of life was not supported.

Several family-related factors should be considered when interpreting our findings. Parental educational levels were lower, and consanguinity was more frequent in the HF group, whereas parental psychiatric diagnoses were more common in the control group. These may have influenced caregiver perceptions, reporting patterns, and children’s developmental environments. Lower parental education and higher rates of consanguinity may contribute to cognitive vulnerability, while parental mental health could affect proxy assessments of child well-being [40]. Even though these differences could not be fully adjusted for, a sensitivity analysis showed that the cognitive gap persisted after separate adjustment for parental education, indicating that it is not explained by parental education alone; residual confounding, nonetheless, cannot be excluded, and parent and child self-reports are best treated as complementary rather than one being more reliable than the other. Although these variables were not the primary focus of our study, they represent important contextual factors and should be addressed in future research with larger samples and longitudinal designs.

Several limitations of this study should be acknowledged. Small sample size reflects the rarity of pediatric end-stage HF but limits statistical power and generalizability. Therefore, subgroup analyses comparing LVAD support, transplant waiting list, and post-transplant patients should be interpreted with caution. The analyses based on gender and sex were not performed due to the small sample size.

The wide age range of the participants relative to the small sample is a further limitation. Although all instruments yield age-standardized scores, children and adolescents across this span differ substantially in cognitive, social, and emotional development, and the sample was too small to stratify the analyses by age or to adjust for it. Age-related heterogeneity may therefore have contributed to the observed variability, and the findings should be interpreted with these limitations in mind.

The cross-sectional design precludes conclusions regarding causal relationships or longitudinal cognitive trajectories across the HF–transplant continuum. Neurocognitive outcomes may reflect disease burden and treatment-stage effects, which this cross-sectional design cannot disentangle; and prospective longitudinal studies are needed to clarify the effects of each treatment.

The WISC-IV assessments could not be completed in all patients due to their clinical conditions, which may have led to selection bias.

Socioeconomic disparities between groups may have influenced neurodevelopmental outcomes and caregiver reporting.

Despite these limitations, this study provides one of the first comprehensive evaluations of neurocognitive function, social cognition, and health-related quality of life across distinct stages of pediatric HF and transplantation, and revealed no statistically detectable additional cognitive difference associated with LVAD support in these small, underpowered subgroup comparisons, from which a treatment effect can be neither confirmed nor excluded. Together, these findings highlight clinically meaningful vulnerabilities that warrant routine multidisciplinary surveillance.

## 6. Conclusions

Caregiver perspectives in assessing everyday functioning should be a part of the evaluation. Our findings emphasize the need for integrated, multidisciplinary care pathways that include routine neuropsychological monitoring, psychiatric follow-up, and individualized educational and psychosocial support for all children with advanced HF.

Despite concerns regarding potential cognitive decline during LVAD support, our findings did not demonstrate worse performance in children with LVAD support, although the treatment-subgroup comparisons were small and underpowered, and the group differences are unadjusted, so neither a specific cause nor the absence of a treatment effect can be established. No statistically detectable subgroup differences were found in this small, exploratory, underpowered sample. The findings require confirmation in larger, ideally multicenter and longitudinal, samples before clinical generalization.

## Figures and Tables

**Table 1:** Sociodemographics of the groups.

	Patient	Control	*p*
**Age (mean ± SD)**	12.739 ± 2.879	13.294 ± 2.468	0.589
	***n*** **(%)**	***n*** **(%)**	
**Sex (male)**	12 (52.2)	9 (52.9)	1.000
**Education**			
**Elementary**	13 (56.5)	10 (58.8)	
**High**	10 (43.5)	7 (41.2)	0.884
**Mother’s Education**			
**University**	2 (8.7)	11 (64.7)	<0.001
**Father’s Education**			
**University**	3 (13.0)	9 (52.9)	0.003
**Mother’s Psychiatric Diagnosis**	3 (13.0)	4 (23.5)	0.326
**Father’s Psychiatric Diagnosis**	1 (4.3)	4 (23.5)	0.144
**Parental Consanguinity**	12 (52.2)	0	<0.001
**Home Education**	14 (60.9)	0	<0.001

**Table 3:** Children’s Depression Inventory (CDI) and Screen for Child Anxiety-Related Emotional Disorders (SCARED) scores of the groups.

Score	Patient Median (Min–Max)	Control Median (Min–Max)	*p* *	r
**CDI**	6.00 (3.00–16.00)	5.00 (1.00–16.00)	0.067	0.34
**SCARED Child Form**				
**Total**	15.00 (8.00–50.00)	13.00 (3.00–31.00)	0.135	0.28
Panic Disorder	4.00 (2.00–12.00)	4.00 (0.00–10.00)	0.539	0.11
Generalized Anxiety Disorder	5.00 (2.00–12.00)	3.00 (0.00–11.00)	0.136	0.28
Separation Anxiety Disorder	3.00 (1.00–11.00)	2.00 (0.00–8.00)	0.286	0.20
Social Anxiety Disorder	3.00 (0.00–10.00)	3.00 (0.00–7.00)	0.349	0.17
School Refusal	1.00 (0.00–3.00)	1.00 (0.00–2.00)	0.703	0.07
**SCARED Parent Form**				
**Total**	17.00 (12.00–35.00)	13.00 (4.00–25.00)	0.001	0.61
Panic Disorder	4.00 (2.00–10.00)	3.00 (0.00–6.00)	0.025	0.41
Generalized Anxiety Disorder	5.00 (3.00–11.00)	4.00 (1.00–4.00)	0.065	0.34
Separation Anxiety Disorder	4.00 (3.00–7.00)	2.00 (0.00–2.00)	0.001	0.62
Social Anxiety Disorder	4.00 (0.00–9.00)	2.00 (0.00–2.00)	0.268	0.20
School Refusal	0.00 (0.00–2.00)	1.00 (0.00–2.00)	0.127	0.25

* Mann–Whitney U test. r = rank-biserial correlation (effect size); 0.1 is small, 0.3 is medium, 0.5 is large. All SCARED-Parent domains were higher in the heart failure group.

**Table 4:** Pediatric QoL Inventory (PedsQL)—Child and Parent Forms (with effect size).

**Child Self-Report**						
	**Patient Mean ± SD**	**Patient Median (Min–Max)**	**Control Mean ± SD**	**Control Median (Min–Max)**	***p***	**Hedges g [95% CI]**
**PHS**	47.87 ± 17.99	46.87 (12.50–87.50)	84.92 ± 14.42	87.50 (50.00–100.00)	0.001 ^1^	−2.19 [−2.98, −1.40]
**EFS**	65.65 ± 14.64	65.00 (30.00–85.00)	80.59 ± 14.78	85.00 (40.00–100.00)	0.003 ^2^	−1.00 [−1.66, −0.33]
**SoFS**	81.53 ± 16.41	85.00 (35.00–100.00)	91.76 ± 15.30	100.00 (40.00–100.00)	0.011 ^1^	−0.63 [−1.27, 0.01]
**ScFS**	59.13 ± 15.64	60.00 (25.00–85.00)	82.65 ± 14.27	90.00 (50.00–100.00)	0.001 ^1^	−1.53 [−2.24, −0.82]
**PHSS**	68.89 ± 10.69	70.00 (51.60–85.00)	85.00 ± 12.84	88.33 (45.00–100.00)	0.001 ^1^	−1.36 [−2.05, −0.66]
**TSS**	61.90 ± 11.15	60.80 (46.70–84.00)	85.03 ± 12.74	90.20 (46.70–100.00)	0.001 ^1^	−1.91 [−2.67, −1.16]
**Parent Proxy Report**						
	**Patient Mean ± SD**	**Patient Median (Min–Max)**	**Control Mean ± SD**	**Control Median (Min–Max)**	***p***	**Hedges g [95% CI]**
**PHS**	56.52 ± 17.47	56.25 (21.87–87.50)	86.58 ± 14.27	93.75 (50.00–100.00)	0.001 ^1^	−1.82 [−2.56, −1.08]
**EFS**	65.43 ± 17.77	65.00 (30.00–95.00)	81.18 ± 14.53	80.00 (45.00–100.00)	0.005 ^2^	−0.94 [−1.60, −0.28]
**SoFS**	78.70 ± 16.32	85.00 (35.00–100.00)	92.94 ± 13.47	100.00 (45.00–100.00)	0.001 ^1^	−0.92 [−1.58, −0.26]
**ScFS**	65.43 ± 15.14	65.00 (40.00–100.00)	81.47 ± 14.66	85.00 (50.00–100.00)	0.002 ^2^	−1.05 [−1.72, −0.38]
**PHSS**	69.55 ± 11.78	66.66 (50.00–93.30)	85.19 ± 11.49	86.66 (60.00–100.00)	0.001 ^2^	−1.32 [−2.01, −0.63]
**TSS**	64.80 ± 11.75	65.20 (45.65–91.00)	85.36 ± 11.20	86.80 (56.50–100.00)	0.001 ^2^	−1.75 [−2.48, −1.01]

^1^ Mann–Whitney U test; ^2^ independent-samples *t* test. Negative g = lower scores in the heart-failure group. PHS: Physical Health Score, ScFS: School Functioning Score, EFS: Emotional Functioning Score, PHSS: Psychosocial Health Summary Score, SoFS: Social Functioning Score, TSS: Total Scale Score.

**Table 5:** (**a**) Comparison of Wechsler Intelligence Scale for Children, Fourth Edition (WISC-IV), index scores (subgroups), between-group and effect size. (**b**) Wechsler Intelligence Scale for Children–Fourth Edition (WISC-IV) subtest scores, effect sizes, and 95% confidence intervals. Subtest *n* varies (17–19) owing to item-level missingness; 8 of 10 subtests differ significantly.

(**a**)												
	**Device-** **Supported (*n* = 6)**	**No Device, Awaiting (*n* = 7)**		**Post-** **Transplant (*n* = 5)**		**Sub-** **Group p**	**Patients, all (*n* = 18)**		**Control (*n* = 17)**	***p***		**Hedges g [95% CI]**
**VCI**	81.33 ± 21.23	83.71 ± 18.16		79.20 ± 13.39		0.980	81.67 ± 17.17		102.24 ± 16.51	0.001		−1.19 [−1.91, −0.47]
**PRI**	82.50 ± 5.13	80.86 ± 18.82		85.20 ± 15.50		0.659	82.61 ± 12.87		100.06 ± 12.25	0.001		−1.21 [−1.93, −0.49]
**WMI**	81.83 ± 18.06	83.29 ± 13.02		75.80 ± 12.66		0.610	80.72 ± 14.27		94.71 ± 16.63	0.009		−0.88 [−1.58, −0.19]
**PSI**	80.83 ± 11.36	78.43 ± 16.00		65.80 ± 15.43		0.270	75.72 ± 15.02		93.94 ± 18.28	0.003		−1.07 [−1.78, −0.36]
**FSIQ**	75.50 ± 16.98	74.43 ± 19.54		70.20 ± 17.51		0.809	73.61 ± 17.23		98.59 ± 17.77	0.001		−1.45 [−2.20, −0.71]
(**b**)												
**Subtest**			***n*** **(pt/ctrl)**		**Hedges g**			**95% CI**			***p***	
Similarities			18/17		−1.41			−2.15 to −0.67			0.001	
Digit Span			19/17		−1.14			−1.84 to −0.43			0.001	
Picture Concepts			17/17		−0.38			−1.06 to 0.30			0.266	
Coding			18/17		−1.14			−1.86 to −0.43			0.002	
Vocabulary			17/17		−0.99			−1.70 to −0.28			0.006	
Comprehension			18/17		−0.72			−1.41 to −0.04			0.036	
Symbol Search			17/17		−0.99			−1.70 to −0.28			0.006	
Letter–Number Seq.			19/17		−0.31			−0.97 to 0.35			0.404	
Matrix Reasoning			18/17		−1.00			−1.70 to −0.29			0.006	
Block Design			19/17		−1.25			−1.97 to −0.54			0.001	

Sub-group p = Kruskal–Wallis across the three treatment subgroups; *p* = patient (all) vs. control (Mann–Whitney U or *t* test). Hedges g = patient vs. control. VCI verbal comprehension; PRI perceptual reasoning; WMI working memory; PSI processing speed; FSIQ full-scale IQ.

**Table 6:** Relationship of WISC-IV index scores with PedsQL–Child and Parent Form (patients).

PedsQL	Child Self-Report					Parent Proxy Report				
	VCI	PRI	WMI	PSI	FSIQ	VCI	PRI	WMI	PSI	FSIQ
**PHS**	−0.20	−0.03	−0.32	−0.32	−0.27	0.33	0.22	0.16	0.02	0.25
**EFS**	0.17	0.34	0.09	0.18	0.22	0.29	0.45	0.12	0.24	0.34
**SoFS**	−0.12	0.10	−0.11	−0.11	−0.12	0.26	0.06	0.48 ^a^	0.24	0.34
**ScFS**	0.44	0.41	0.31	0.20	0.39	0.52 ^a^	0.46	0.55 ^a^	0.43	0.51 ^a^
**PHSS**	0.27	0.36	0.15	0.12	0.25	0.56 ^a^	0.49 ^a^	0.55 ^a^	0.45	0.60 ^a^
**TSS**	0.02	0.25	−0.08	−0.05	0.01	0.57 ^a^	0.38	0.55 ^a^	0.38	0.56 ^a^

Spearman correlations (ρ) of WISC-IV index scores with PedsQL domain scores in patients (*n* = 18). ^a^ nominally *p* < 0.05, but no coefficient remained significant after Benjamini–Hochberg FDR correction (all adjusted *p* ≥ 0.16); coefficients are exploratory. VCI, verbal comprehension; PRI, perceptual reasoning; WMI, working memory; PSI, processing speed; FSIQ, full-scale IQ; PHS/ EFS/ SoFS/ ScFS/ PHSS/ TSS, PedsQL physical/ emotional/ social/ school/ psychosocial-summary/ total scores.

**Table 7:** Relationship of WISC–IV index scores with PedsQL–Child and Parent Form (control).

PedsQL	Child Self-Report					Parent Proxy Report				
	VCI	PRI	WMI	PSI	FSIQ	VCI	PRI	WMI	PSI	FSIQ
**PHS**	0.26	0.10	0.30	−0.06	0.21	0.27	0.27	0.61 ^a^	0.07	0.46
**EFS**	−0.03	−0.05	0.40	0.12	0.21	0.10	0.10	0.28	−0.27	0.06
**SoFS**	0.18	0.19	0.37	−0.09	0.10	0.38	0.17	0.63 ^a^	0.04	0.38
**ScFS**	0.08	−0.05	0.17	−0.20	0.00	0.27	0.21	0.63 ^a^	0.05	0.37
**PHSS**	0.10	0.03	0.39	−0.11	0.12	0.36	0.21	0.65 ^a^	0.03	0.41
**TSS**	0.20	0.13	0.42	−0.10	0.21	0.01	0.10	0.41	−0.10	0.13

Spearman correlations (ρ) of WISC-IV index scores with PedsQL domain scores, controls (*n* = 17). ^a^ nominally *p* < 0.05, but no coefficient remained significant after Benjamini–Hochberg FDR correction (all adjusted *p* ≥ 0.15); coefficients are exploratory. The only nominal associations were between parent-reported health-related quality of life and working memory (WMI). PHS: Physical Health Score, ScFS: School Functioning Score, EFS: Emotional Functioning Score, PHSS: Psychosocial Health Summary Score, SoFS: Social Functioning Score, TSS: Total Scale Score, PRI: Perceptual Reasoning Index, VCI: Verbal Comprehension Index, WMI: Working Memory Index, PSI: Processing Speed Index, FSIQ: Full Scale IQ.

## Data Availability

The data underlying this article were provided by Ege University Hospital by permission. Data will be shared on request to the corresponding author with permission of Ege University Hospital. The data are not publicly available due to data protection policy of our Minister of Health.
